# Immune Escape-Related Gene *NXT1* as a Potential Prognostic and Therapeutic Target in Hepatocellular Carcinoma

**DOI:** 10.5152/tjg.2025.24193

**Published:** 2025-08-25

**Authors:** Jia Guo, Xin Tong, Songtao Liu, Feng Liu, Yachao Qu, Ren Li, Xuli Bao

**Affiliations:** 1 Hepatology and Cancer Biotherapy Ward, Beijing You’an Hospital, Capital Medical University, Beijing, China; 2 Department of Clinical Laboratory, Tianjin Medical University Cancer Institute and Hospital, National Clinical Research Center for Cancer, Key Laboratory of Cancer Prevention and Therapy, Tianjin’s Clinical Research Center for Cancer, Tianjin, China; 3 Department of Clinical Laboratory, Tianjin Stomatological Hospital, Nankai University School of Medicine, Tianjin Key Laboratory of Oral and Maxillofacial Function Reconstruction, Tianjin, China; 4 Department of Head and Neck Surgery, Shanxi Provincial Cancer Hospital/Shanxi Hospital Cancer Hospital of Chinese Academy of Medical Sciences, Taiyuan, China; 5 Department of Respiratory and Critical Care Medicine, Beijing You’an Hospital, Capital Medical University, Beijing, China

**Keywords:** Hepatocellular carcinoma, immune escape, immunotherapy, NXT1

## Abstract

**Background/Aims:**

Hepatocellular carcinoma (HCC) constitutes approximately 85% of liver cancers. This study aimed to investigate the role of immune escape-related genes (IEGs) in HCC patients and analyze their relationship with prognosis and immunotherapy, thereby providing a reference for further clinical treatment.

**Materials and Methods:**

Datasets were collected from The Cancer Genome Atlas (TCGA) and Gene Expression Omnibus databases. Differential expression analysis was conducted to identify differentially expressed IEGs between normal and HCC tissues. Expression, survival, mutational, and immune profiles of a hub gene nuclear transport factor 2 like export factor 1 (*NXT1*) were evaluated. Validation of NXT1 expression in adjacent normal and HCC tissues was carried out using RT-qPCR and western blot assays. Next, CCK-8, wound healing, and transwell assays were conducted to evaluate the biological function of *NXT1* in HCC cell lines.

**Results:**

Analysis of the TCGA-LIHC and GSE164359 datasets revealed that *NXT1 *was notably elevated in HCC tissues compared to adjacent normal tissues, a finding validated through RT-qPCR and western blot assays. Meanwhile, high levels of *NXT1* were associated with an unfavorable prognosis of HCC patients. Mutational analysis indicated a higher incidence of TP53 mutations in the *NXT1* high-expression group relative to the* NXT1* low-expression group. HCC patients with high *NXT1* expression demonstrated an increased proportion of M0 macrophages and regulatory T cells (Tregs) and a decreased proportion of M1 macrophages. Furthermore, deficiency of *NXT1* significantly suppressed HCC cell viability, migration, and invasion.

**Conclusion:**

Collectively, *NXT1* may serve as a valuable prognostic marker and a potential therapeutic target for HCC.

**Main Points**

- The gene *NXT1* is highly expressed in hepatocellular carcinoma samples.
- Hepatocellular carcinoma patients with high expression of NXT1 have a relatively poor prognosis.
- NXT1 plays oncogenic roles in hepatocellular carcinoma.

## Introduction

Hepatocellular carcinoma (HCC), comprising approximately 85% of primary liver cancer (PLC) cases, is the most prevalent type of PLC and is associated with a high rate of mortality.^1^ Early HCC can be treated with surgical resection, local percutaneous ablation, and liver transplantation.^2^ Unfortunately, many patients are diagnosed in the late stage with poor prognosis.^3^ Due to primary and acquired resistance, most advanced HCC patients cannot obtain long-term benefits from systemic treatment.^4^

In recent years, the introduction of immunotherapy into clinical practice has changed the pattern of cancer treatment and greatly improved the prognosis of cancer patients.^5^ While current treatment guidelines for HCC do not recommend immunotherapy for early or intermediate-stage disease, immune checkpoint inhibitors (ICIs) that target the CTLA-4 or PD-1/PD-L1 axis, such as ipilimumab and durvalumab, have emerged as first-line standard therapies for advanced HCC.^6^ These agents have shown promising clinical efficacy in patients with advanced HCC.^7^ Studies have pointed out that the existence of the immune escape (IE) phenomenon enables tumor cells to escape from immune clearance, thereby reducing the effectiveness of immunotherapy.^8^^,^^9^ Increasing evidence shows that various cells in the tumor microenvironment (TME) are involved in the occurrence of tumor IE.^10^ Although the mechanism of tumor IE is complex and is still not well understood, it is worth noticing that some scholars have found that liver cancer cells may enhance the ability to escape immune surveillance and attack by low oxygen metabolism, abnormal expression of self-antigens, and occurrence of epithelial-mesenchymal transition.^11^^,^^12^ Therefore, a comprehensive analysis of immune escape-related genes (IEGs) expression in the TME of HCC helps formulate reasonable treatment plans. In recent years, the extensive application of bioinformatics technology in tumor immunotherapy can help explore the association between tumors and immune cells more deeply. The objective of this study was to comprehensively analyze IEGs expression levels in HCC patients and their relationship with prognosis and immunotherapy, providing a reference for further clinical management.

## Materials and Methods

### Data Acquisition

Gene expression profiling data of 374 liver HCC (LIHC) samples and 50 adjacent normal samples were downloaded from The Cancer Genome Atlas (TCGA) database, as well as clinical information of 368 samples of cancerous tissue that possess comprehensive data regarding patient survival, which can be found in Table 1. Additionally, Mutation Annotation Format (MAF) files of 364 liver cancer patients were obtained for further analysis. Furthermore, the International Cancer Genome Consortium-Liver Cancer-RIKEN-Japan (ICGC-LIRI-JP) dataset was downloaded from the International Cancer Genome Consortium (ICGC), which encompassed a cohort of 260 individuals with HCC, out of which survival data was fully available for 231 patients (Table 2). Samples from the TCGA-LIHC and ICGC-LIRI-JP datasets were analyzed using the Illumina HiSeq platform to obtain raw data. Subsequently, STAR software version 2.7.3 (Cold Spring Harbor Laboratory; New York, USA) was employed to align the raw data with the reference genome, allowing for gene expression matrix generation.

The GSE164359 dataset was also obtained from the Gene Expression Omnibus (GEO) database, which involved a total of 20 primary tumor specimens and 27 recurrent tumor specimens. This dataset was analyzed using the Illumina HiSeq 4000 platform. Next, STAR software (version 2.7.3) was employed to align the raw data with the reference genome, allowing for gene expression matrix generation.

Besides, 182 IEGs were sorted out according to the published literature (Supplementary Table 1).^13^^,^^14^

### Differential Gene Expression and Functional Enrichment Analysis

The study was performed differential expression analysis using the “limma” package in R language (4.1.0),^15^ with |log_2_FC| > 0.5 and *P*-value < .05 as a benchmark for filtering differentially expressed genes (DEGs).

Based on DEGs obtained, Gene Ontology (GO, including biological process (BP), molecular function (MF), and cellular component (CC)) terms and Kyoto Encyclopedia of Genes and Genomes (KEGG) pathways analyses were conducted using the “clusterProfiler” package.^16^ A *P*-value less than .05 was considered as the significant threshold.

### Survival Analysis

The approach for estimating the overall survival rates over a 10-year period among various groups was facilitated by utilizing the “survival” and “survminer” packages, which are based on the Kaplan–Meier algorithm. Furthermore, the log-rank test was employed to assess the statistical significance of disparities in survival rates among the distinct groups.

### Mutation Analysis

The MAF files of somatic mutations were obtained from the TCGA database, and then they were input into the “maftools” package for analysis and visualization.

### Infiltration Analysis of Immune Cells

The relative abundance of 22 immune cell types within the samples was determined using CIBERSORT.^17^ Subsequently, the immune scores of these samples were quantified by employing the “estimate” package available in R.

### Gene Prioritization and Biomarker Comparison

The analysis focused on the prioritization of genes through the biomarker assessment component of the Tumor Immune Dysfunction and Exclusion (TIDE) framework, involving an examination of the effects of immune checkpoint blockade (ICB) therapy and the phenotypic consequences of gene knockouts as determined by Clustered Regularly Interspaced Short Palindromic Repeats screening. To quantify the influence of gene expression on patient survival within the dataset of ICB-treated patients, *Z*-scores derived from Cox proportional hazards regression were utilized.

In further analysis, the overall predictive accuracy of treatment response and patient overall survival (OS) was compared across various cancer types with 7 established biomarkers that reflect tumor immune status. These biomarkers included T-cell clonality (T.Clonality), B-cell clonality (B.Clonality), TIDE score, the estimated score for microsatellite instability (MSI), tumor mutation burden (TMB), the expression levels of the cell surface molecule cluster of differentiation 274 (CD274), and the presence of interferon-γ (IFNG).

### Clinical Samples

Tumor tissues and adjacent nontumor tissues were obtained from patients with HCC who underwent surgical resection (n = 10) at Shanxi Provincial Cancer Hospital/Shanxi Hospital Cancer Hospital of Chinese Academy of Medical Sciences. The general clinical information of patients is shown in Supplementary Table 2. This research adhered to the ethical standards outlined in the Declaration of Helsinki and received approval from the Ethics Committee of Shanxi Provincial Cancer Hospital/Shanxi Hospital Cancer Hospital of Chinese Academy of Medical Sciences (approval no: KY2024010; date: January 6, 2024). Informed written consent was acquired from all participating patients.

### Reverse Transcription Quantitative Real-Time Polymerase Chain Reaction

The TriQuick Reagent (Solarbio) was employed for isolating total RNA. After that, the isolated RNA underwent reverse transcription to synthesize cDNA utilizing an Evo M-MLV kit (Accurate Biology). Subsequently, the SYBR Master Mix kit (CWBIO) was used for measuring nuclear transport factor 2 like export factor 1 (*NXT1*) mRNA expression. Glyceraldehyde-3-Phosphate Dehydrogenase (*GAPDH*) served as an internal control. The primer sequences used for quantitative polymerase chain reaction are listed in Supplementary Table 3.

### Western Blot Assay

Proteins were separated by 10% SDS-PAGE gels, followed by electrotransfer onto polyvinylidene difluoride membranes. The membranes were blocked and then blotted with primary and secondary antibodies. Subsequently, immunoreactive bands were detected by electrochemiluminescence reagents (Beyotime). The primary antibodies used in this study were as follows: anti-NXT1 and anti-GAPDH antibodies were obtained from Proteintech.

### Cell Culture and Transfection

Transformed human liver epithelial-2 (THLE-2) cells (IMMOCELL) were cultured in a complete Bronchial epithelial growth medium. HCCLM3 (Procell) and Huh-7 (Procell) cells were cultured in a Dulbecco's Modified Eagle Medium (DMEM) medium containing 10% FBS and 1% P/S. HepG2 cells were cultured in Minimum Essential Medium (MEM) medium containing 10% FBS and 1% P/S. All cell lines were maintained at 37°C in a humidified atmosphere containing 5% CO_2_.

HCCLM3 cells were transfected with siRNA negative control (siRNA NC), *NXT1* siRNA1, *NXT1* siRNA2, and *NXT1* siRNA3 using the Transfection Kit. The siRNA sequences are summarized in Supplementary Table 4.

### Cell Counting Kit-8 Assay

HCCLM3 cells (5 × 10^3^ cells) were seeded into a 96-well plate at 37°C. Next, 10 μL of CCK-8 solution (Beyotime) was added to each well, and cells were then incubated for 2 hours. The absorbance for each well was assessed using a microplate reader at 450 nm.

### Wound Healing Assay

HCCLM3 cells were plated into 12-well plates at 37°C overnight. Next, a scratch was made in the center of each well using a sterilized pipet tip. After 0, 24, or 48 hours of incubation, photographs were taken using a light microscope.

### Transwell Assay

HCCLM3 cells, suspended in a serum-free medium, were seeded into the upper chamber of the Transwell and incubated with a medium containing 10% FBS in the bottom chamber. After 24 hours of incubation, the cells on the bottom side of the filter were stained with 0.1% crystal violet. Images were subsequently obtained using a light microscope. For cell invasion assays, the filter was pre-coated with Matrigel (Corning) before conducting the experiments.

### Statistical Analysis

Data are shown as means ± SDs. The comparison between 2 groups was conducted using the t-test, whereas one-way ANOVA was employed to assess differences among multiple groups. A *P*-value of less than .05 was deemed significant.

## Results

### Detection of Differentially Expressed Immune Escape-Related Genes in Hepatocellular Carcinoma

**Figure 1.**
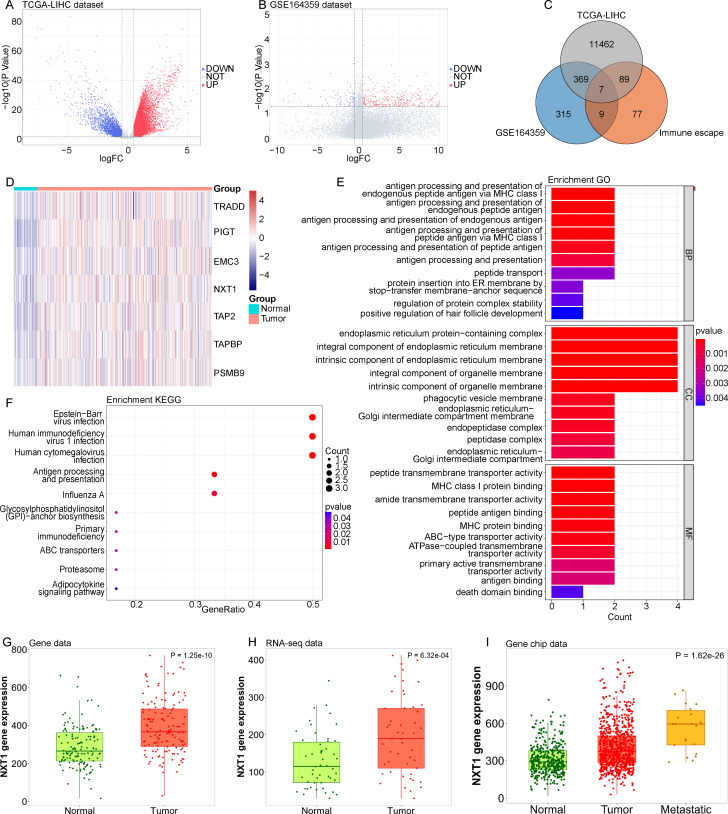
Identification of immune escape-related genes in hepatocellular carcinoma. (A, B) Differential gene expression between tumor samples and normal samples in TCGA-LIHC and GSE164359 datasets. (C) Venn diagram demonstrates the intersections of genes among TCGA-LIHC data, GSE164359 data, and 182 IEGs. (D) Heatmap of the expression of 7 intersection genes in normal and LIHC tissues in the TCGA-LIHC dataset. (E, F) GO and KEGG enrichment analysis of these 7 intersection genes. Top 10 significantly enriched GO terms (BP, CC, MF) and KEGG pathways. (G, H) *NXT1* expression in normal and HCC tissue was analyzed using the TNMplot database from gene data and RNA-seq data. (I) *NXT1* expression in normal, HCC tissue, and metastatic tissue were analyzed using the TNMplot database from gene chip data.

We conducted a differential analysis of tumor versus normal tissues within the TCGA-LIHC cohort, identifying 11 927 DEGs (Fig. 1). Additionally, a differential analysis was performed between cancer samples in situ and recurrent cancer samples using the GSE164359 dataset, identifying 700 DEGs (Fig. 1). A cross-analysis was then performed between these 2 sets of DEGs and 182 IEGs (Supplementary Table 1), resulting in the identification of 7 overlapping genes (Fig. 1). Notably, these 7 genes exhibited high expression levels in the tumor samples from the TCGA-LIHC dataset (Fig. 1). The analysis of the functional roles of these 7 genes revealed that they were significantly enriched in 91 GO terms (Fig. 1, Supplementary Table 5), and in 10 KEGG Pathways (Fig. 1, Supplementary Table 6).

Among the 7 genes, *NXT1 *exhibited increased expression levels in tumor specimens from both TCGA and GEO datasets using the online database TNMplot (Fig. 1). Furthermore, in the GEO database, metastatic samples displayed a significant elevation in *NXT1* expression compared to both the tumor and normal tissue samples (Fig. 1). Hence, *NXT1* was selected as the target IEG.

### The Expression and Survival Analysis of NTX1 in Hepatocellular Carcinoma Patients

**Figure 2.**
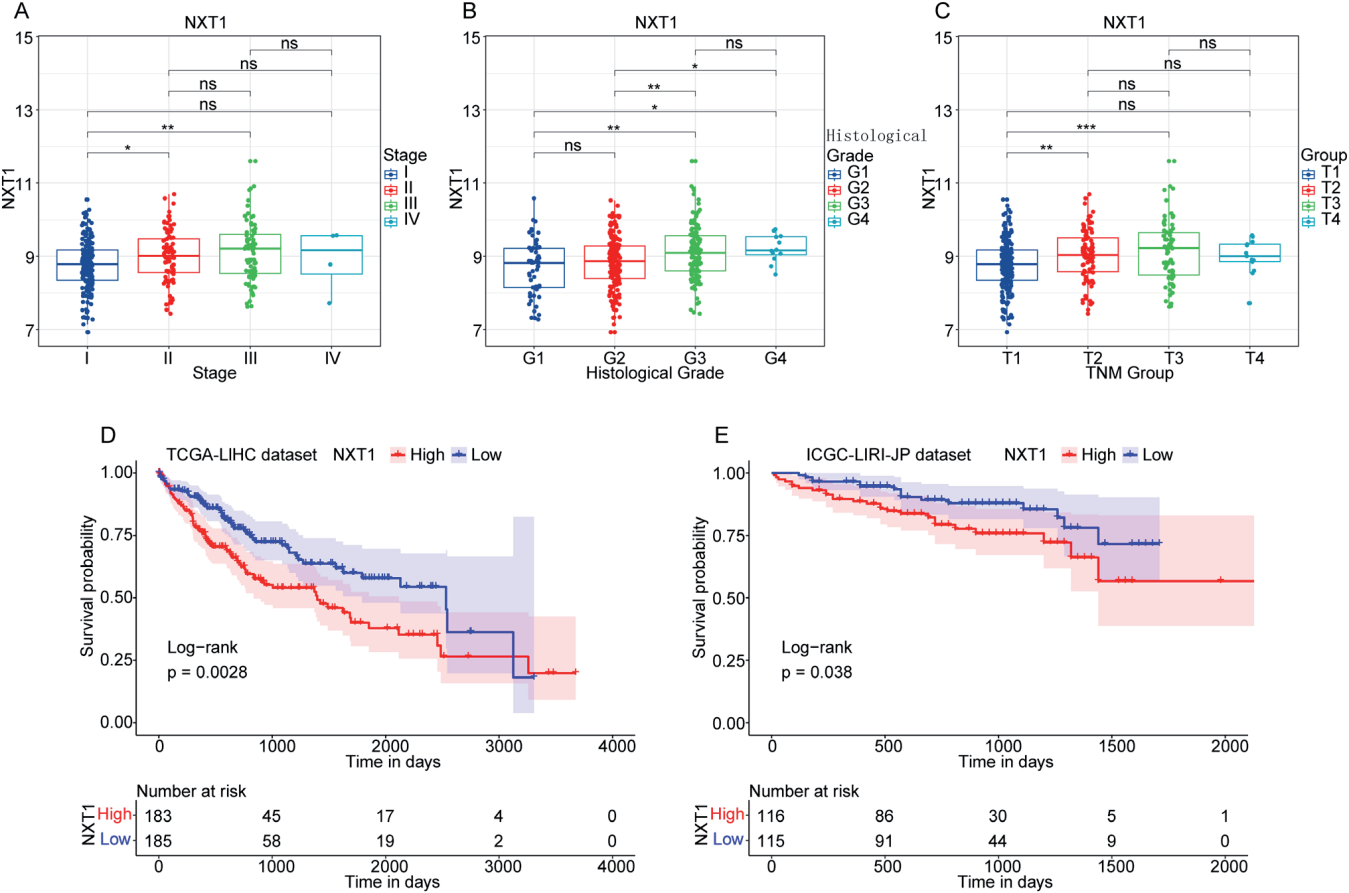
The expression and survival analysis of *NXT1* in hepatocellular carcinoma patients. (A) The expression of *NXT1* in HCC patients at different clinical stages (I, II, III, IV) in the TCGA-LIHC dataset. (B) The expression of *NXT1* in HCC patients at different histological grades (G1-G4) in the TCGA-LIHC dataset. (C) The expression of *NXT1* in HCC patients at the TNM staging system (T1-T4) in the TCGA-LIHC dataset. (D-E) KM survival analysis between the *NXT1*^high^ group and the *NXT1*^low^ group in TCGA-LIHC and ICGC-LIRI-JP datasets.

In the TCGA-LIHC dataset, which included 368 samples with comprehensive survival data, the levels of NXT1 expression were assessed across various clinical stages of the disease, ranging from I to IV, using the available clinical details. The findings revealed that *NXT1* expression varied significantly when comparing stage I to both stage II and stage III, with a progressive rise in expression observed (Fig. 2). In addition, *NXT1* expression was analyzed in different histological grades (G1-G4), revealing significant differences among various group pairings including G1 with G3, G1 with G4, G2 with G3, and G2 with G4, all of which demonstrated increasing expression levels (Fig. 2). Furthermore, the expression of *NXT1* was analyzed in the TNM (Tumor, Node, and Metastasis) staging system (T1-T4), and the results showed significant differences between T1 and T2, as well as between T1 and T3, with an increasing expression level (Fig. 2).

Survival analyses were conducted on HCC datasets from both TCGA-LIHC and ICGC-LIRI-JP, stratifying the samples into high-*NXT1* (*NXT1*^high^) and low-*NXT1* (*NXT1*^low^) groups based on the median expression values of *NXT1* (median value = 8.906890596 for the TCGA-LIHC cohort; median value = 57129 for the ICGC-LIRI-JP cohort). The results showed that individuals in the *NXT1*^high^ group had a relatively poor prognosis in comparison to those in the *NXT1*^low^ group (Fig. 2).

### Variations in Mutation Status Between *NXT1*^high^ Group and *NXT1*^low^ Group

**Figure 3.**
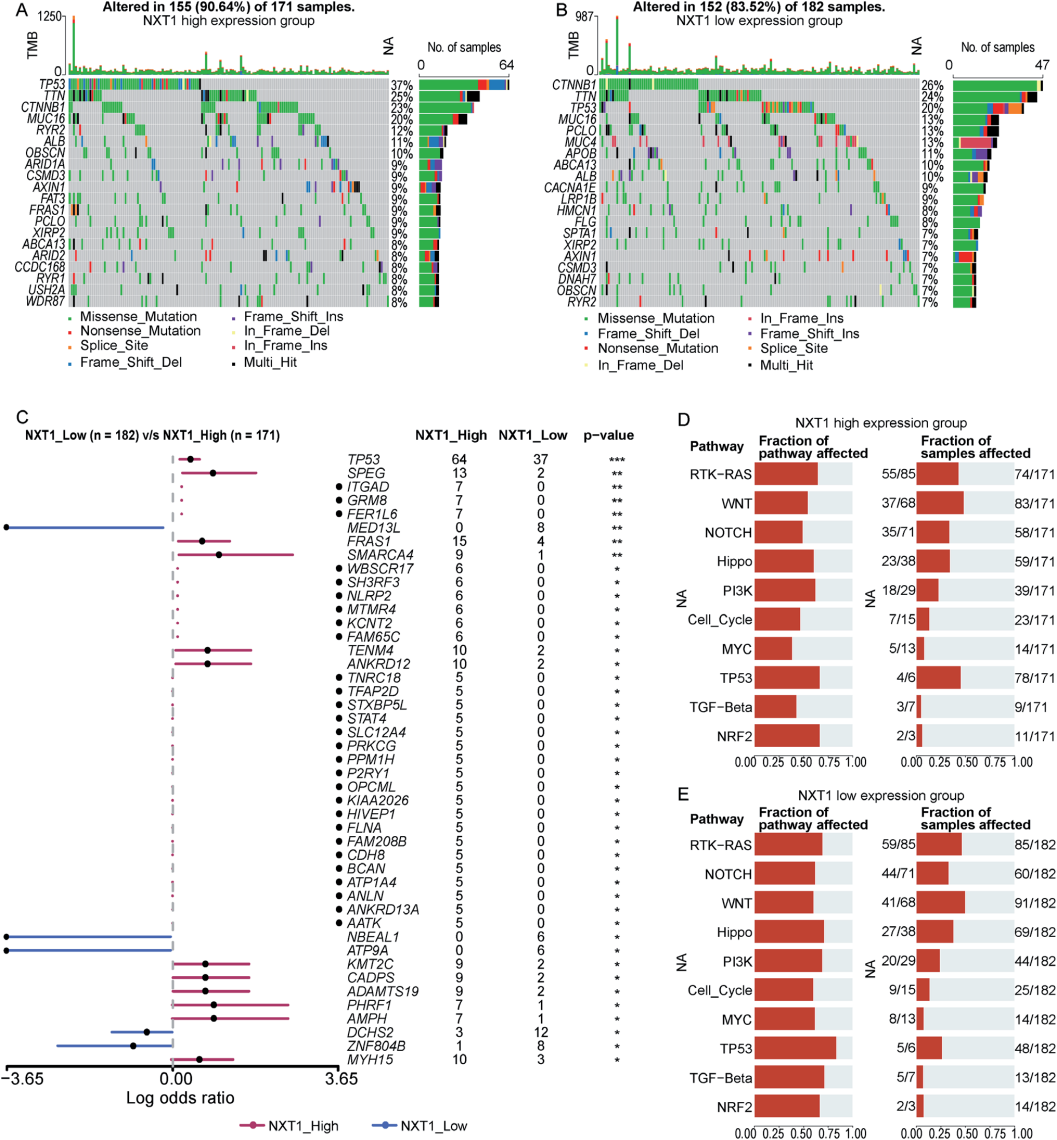
Mutation analysis in high and low *NXT1* expression groups. (A-B) Mutation analysis in *NXT1*^high^ group and *NXT1*^low^ group. (C) Differences in mutation genes between *NXT1*^high^ group and *NXT1*^low^ group. (D-E) The ratios of gene mutation in 10 tumor-related pathways in *NXT1*^high^ group and *NXT1*^low^ group.

To gain insights into the underlying genetic mechanisms that promote the growth and advancement of HCC, somatic mutations, and TMB were analyzed in HCC. Using somatic mutation data from the TCGA-LIHC dataset, differences were observed in TMB levels between the *NXT1*^high^ group and the *NXT1*^low^ group. The mutation analysis showed that the incidence of *TP53* mutations (37%) was the highest in the *NXT1*^high^ group (Fig. 3), while the highest incidence in the *NXT1*^low^ group was *CTNNB1* mutations (26%) (Fig. 3). Additionally, significant differences in mutations of 45 genes’ mutations between *NXT1*^high^ group and *NXT1*^low^ group were found (*P* < .05, Fig. 3). Among the 10 tumor-related pathways examined, the fraction of samples affected by mutations in genes from the *TP53* pathway was higher in *NXT1*^high^ group (Fig. 3) compared to *NXT1*^low^ group (Fig. 3).

### The Relationship Between *NXT1* and Chemokines/Chemokine Receptors

**Figure 4.**
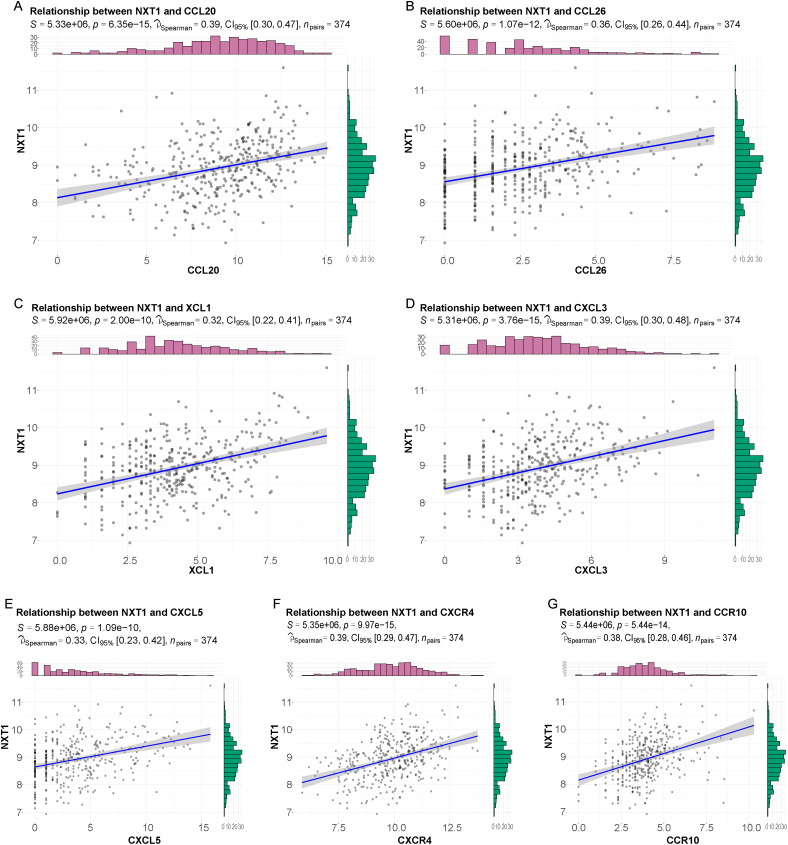
Relationship between *NXT1* and chemokines/chemokine receptors. (A-G) Correlation analysis of *NXT1* expression level and 7 most significant chemokines/chemokine receptors.

Chemokines and their receptors are recognized for their significant contributions to the pathology of cancer progression and metastasis. A correlation analysis was conducted between *NXT1* and 41 chemokines as well as 18 chemokine receptors. Based on the criterion of *P* < .05 and |correlation| > 0.3, significant positive correlations were found between *NXT1* and the chemokines *CCL20*, *CCL26*, *XCL1*, *CXCL3*, and *CXCL5* (Fig. 4). Additionally,* NXT1* also showed significant positive correlations with the chemokine receptors *CCR10 *and *CXCR4 *(Fig. 4).

### Immune Landscapes of High and Low *NXT1* Expression Groups

**Figure 5.**
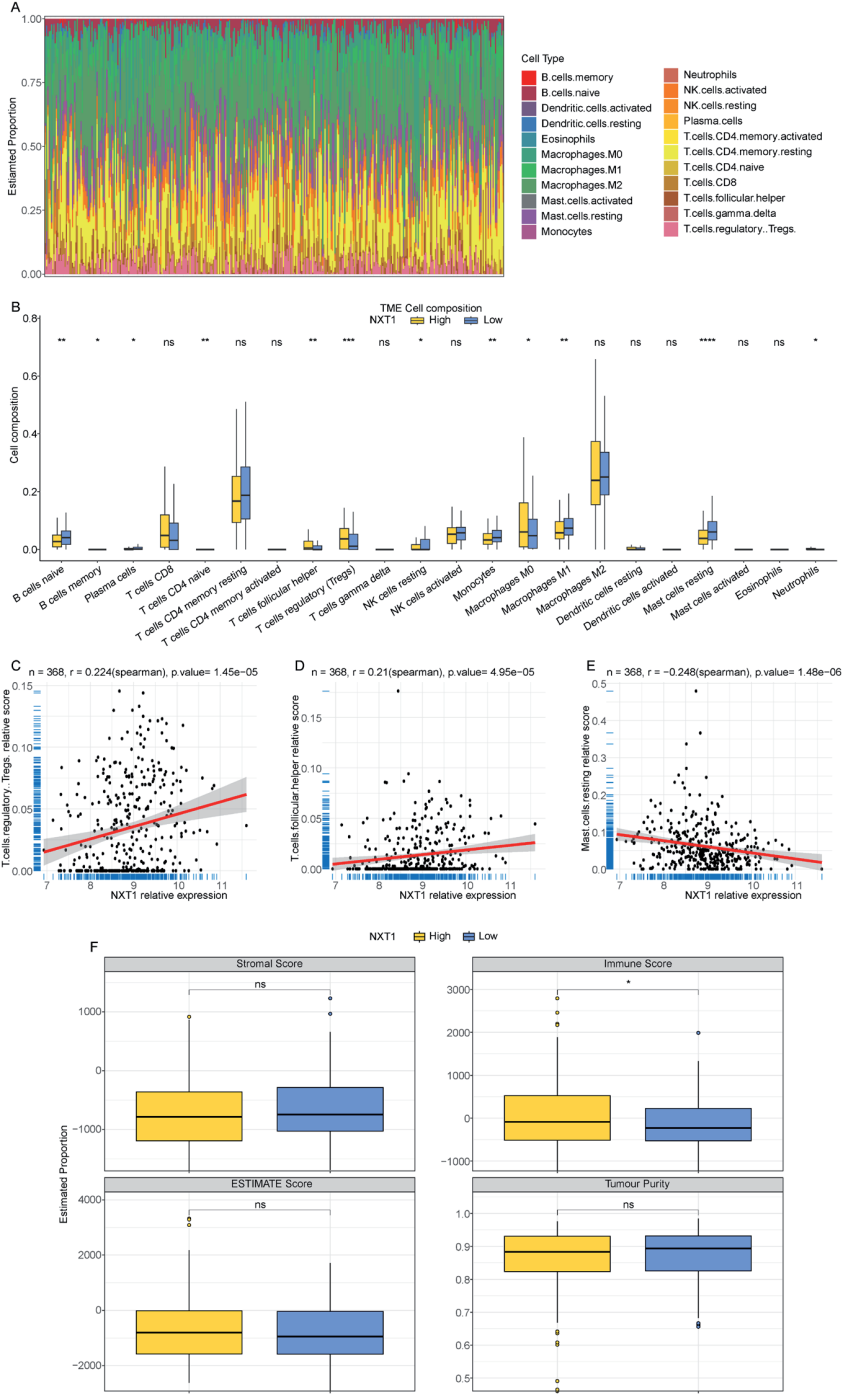
Immune infiltration analysis of hepatocellular carcinoma samples. (A) Relative abundance of 22 types of immune cells across all samples. (B) The infiltration ratios of 22 types of immune cells in the *NXT1*^high^ group and the *NXT1*^low^ group. (C-E) Correlation analysis between* NXT1* expression and Tregs, follicular helper T cells, and resting mast cells. (F) ImmuneScore, ESTIMATEScore, StromalScore, and TumorPurity in *NXT1*^high^ group and *NXT1*^low^ group.

Leveraging the CIBERSORT analytical tool, the proportional presence of 22 distinct immune cell populations within HCC samples obtained from the TCGA dataset was determined (Fig. 5). Infiltration ratios of 12 types of immune cells (naive B cells, memory B cells, plasma cells, naive CD4 T cells, follicular helper T cells (Tfh), regulatory T cells (Tregs), resting natural killer (NK) cells, monocytes, macrophages M0, macrophages M1, resting mast cells, neutrophils) exhibited substantial disparities when comparing the *NXT1*^high^ and *NXT1*^low^ groups (Fig. 5). Additional examination of the relationship between *NXT1* expression levels and the 12 immune cells that showed significant differences revealed a substantial positive association between *NXT1* and Tregs, as well as Tfhs (*P*-value < .05) (Fig. 5,), and a significant negative correlation with resting mast cells (*P*-value < .05) (Fig. 5).

Moreover, it was found that the ImmuneScore was significantly elevated in the *NXT1*^high^ group compared to the *NXT1*^low^ group, suggesting that *NXT1* may be a viable target for immunotherapeutic approaches; whereas ESTIMATEScore, TumorPurity, and StromalScore did not exhibit any significant variance between the *NXT1*^high^ and *NXT1*^low^ groups (Fig. 5). Collectively, these findings indicate a distinct immune landscape between the *NXT1*^high^ and *NXT1*^low^ groups, which indirectly supports the potential impact of *NXT1* on the tumor immune microenvironment in HCC.

### *NXT1* was Valuable in Hepatocellular Carcinoma Immunotherapy

**Figure 6.**
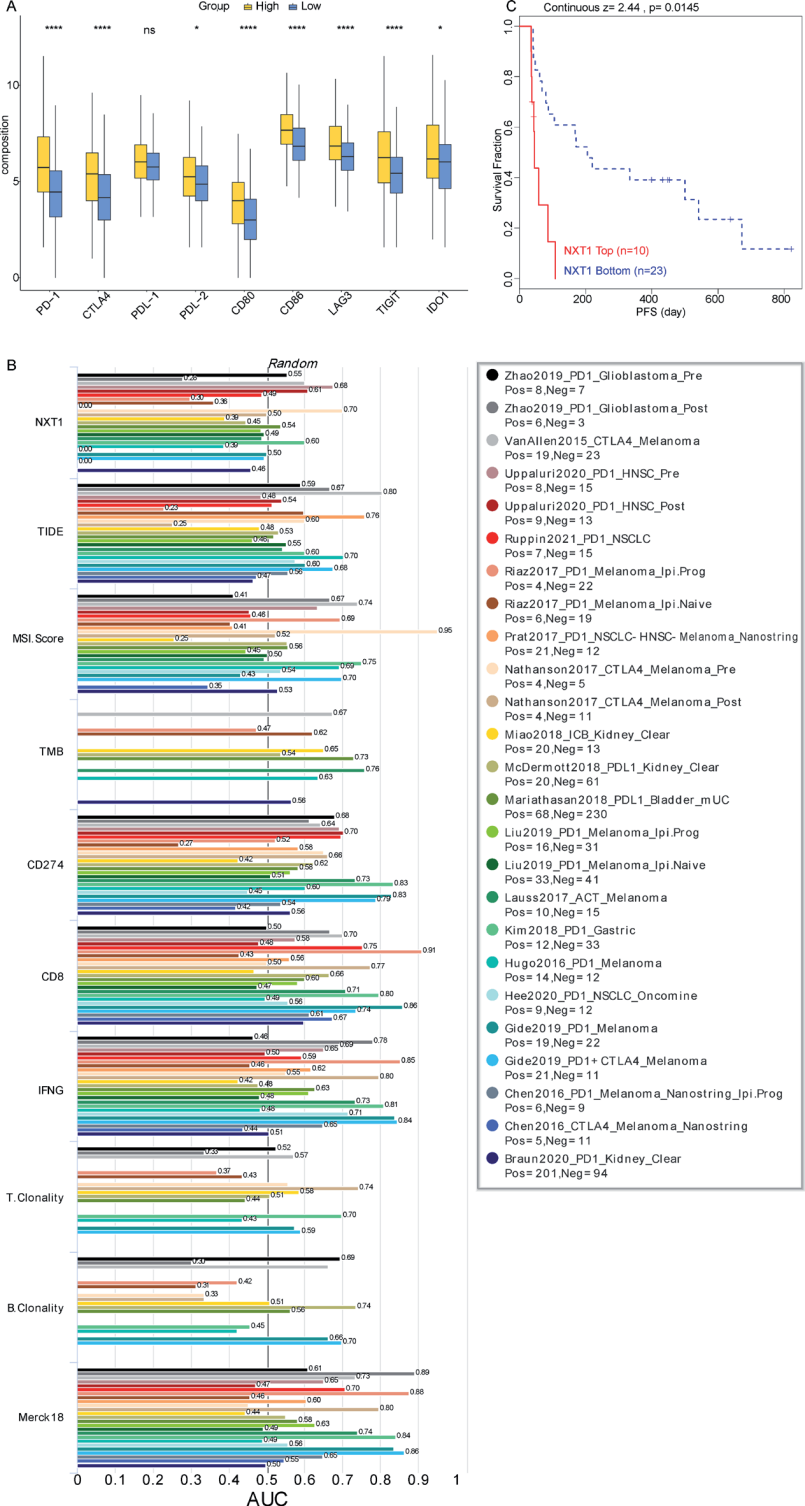
Analysis of immune therapy response and immune checkpoint expression. (A) Expression of 9 immune checkpoint genes in *NXT1*^high^ group and *NXT1*^low^ group. (B) Correlation of *NXT1* with standardized cancer immune evasion biomarkers in the ICB datasets. (C) KM survival curves of *NXT1* predicting ICB treatment in the Miao2018_ICB dataset.

To further determine *NXT1*’s value on immunotherapy, expression levels of 9 immune checkpoints *PDCD1* (PD-1), *CTLA4*, *TIGIT*, *CD274* (PDL-1), *PDCD1LG2* (PDL-2), *IDO1*, *CD80*, *CD86*, and *LAG3* were analyzed between the *NXT1*^high^ group and the *NXT1*^low^ group. The findings revealed that the levels of expression for 8 immune checkpoints—*PDCD1* (PD-1), CTLA4, *PDCD1LG2* (PDL-2), CD80, CD86, LAG3, TIGIT, and IDO1—were significantly higher in the *NXT1*^high^ group compared to *NXT1*^low^ group (Fig. 6).

Additionally, *NXT1* was compared with some standardized biomarkers and evaluated the prognostic value. Among the 25 ICB datasets, it was found that *NXT1* alone had an area under the receiver-operating characteristic curve (AUC) of ≥ 0.5 in 9 of the 25 ICB sub-cohorts, indicating a more robust predictive capacity compared to TMB (8) and B. Clonality (7), but lower than TIDE score, MSI score, CD27A, IFNG, CD8, T.Clonality, and Merck18 (Fig. 6). In the Miao2018_ICB dataset, which included immunotherapy clinical response information, patients with high *NXT1* expression had a significantly worse prognosis (Fig. 6).

### Downregulation of *NXT1* Suppressed the Migration and Invasion of Hepatocellular Carcinoma Cells

**Figure 7.**
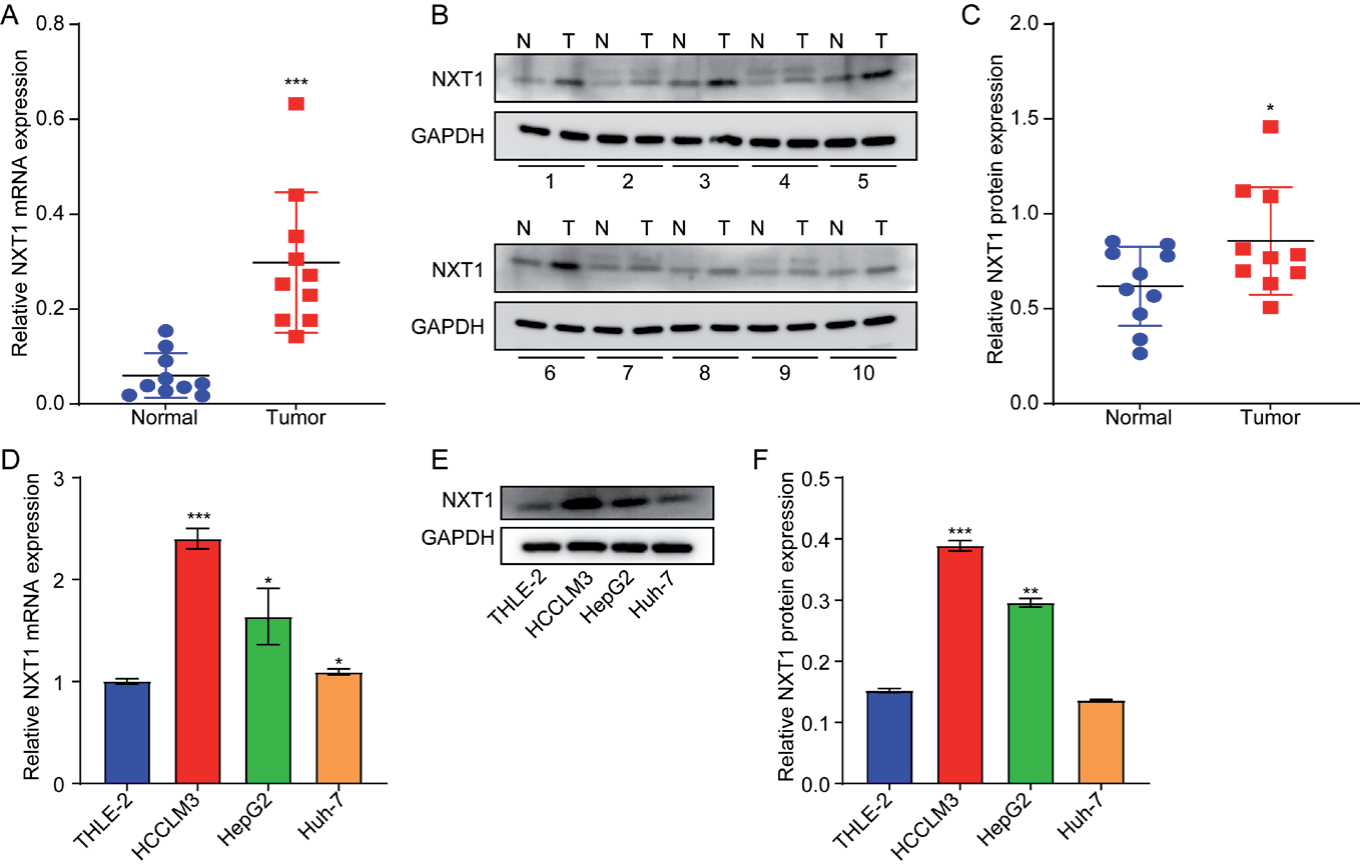
NXT1 expression is upregulated in hepatocellular carcinoma tissues and hepatocellular carcinoma cell lines. (A, B, C) RT-qPCR and western blot analyses of NXT1 levels in HCC tissues (n = 10) and adjacent normal tissues (n = 10). *P < .05, ***P < .01 vs Normal group. (D, E, F) RT-qPCR and western blot analyses of NXT1 levels in THLE-2, HCCLM3, HepG2, and Huh7 cells. *P < .05, **P < .01, ***P < .01 vs THLE-2 group.

To validate *NXT1* expression in HCC, *NXT1 *levels were evaluated in tumor tissues and adjacent normal samples. As illustrated in Fig. 7A-7C, the mRNA and protein levels of NXT1 were notably elevated in tumor tissues compared to adjacent normal samples. Meanwhile, NXT1 levels were markedly increased in HCC cell lines, HCCLM3 and HepG2, when compared to THLE-2 cells, with HCCLM3 cells exhibiting the highest levels of NXT1 expression (Fig. 7D-7F).

**Figure 8.**
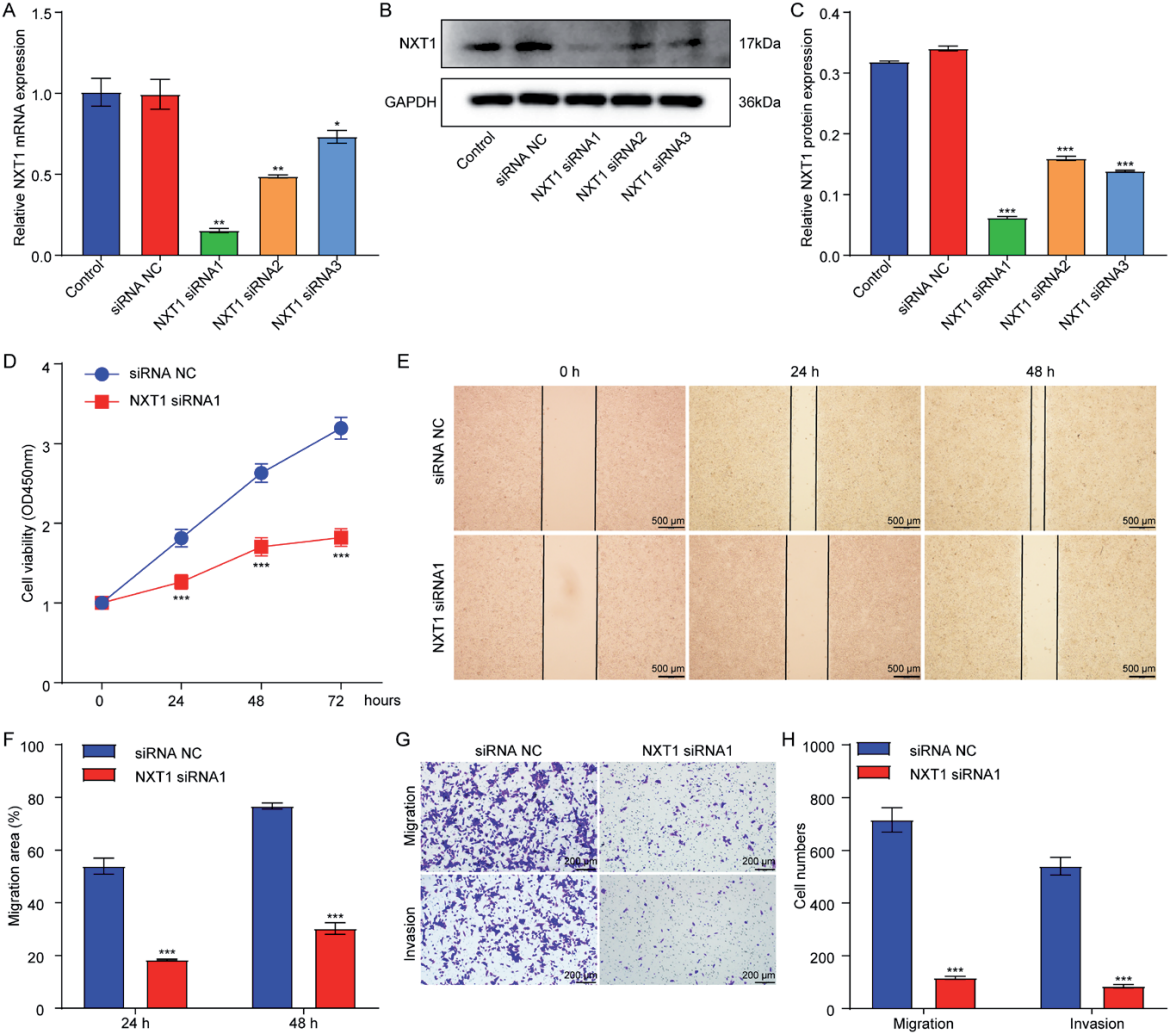
Downregulation of *NXT1* suppressed the migration and invasion of hepatocellular carcinoma cells. (A, B, C) HCCLM3 cells were transfected with siRNA NC, *NXT1* siRNA1, *NXT1* siRNA2, and *NXT1* siRNA3. Reverse transcription quantitative polymerase chain reaction and western blot assays were applied to assess NXT1 mRNA and protein levels in transfected HCCLM3 cells. (D) HCCLM3 cells were transfected with siRNA NC and *NXT1 *siRNA1. CCK-8 assay was conducted to evaluate cell viability. (E, F) Wound healing assay was employed to determine cell migration. (G, H) Transwell assay was conducted to assess cell migration and invasion. *P < .05, **P < .01, ***P < .01 vs siRNA NC group.

Next, to explore the functional role of *NXT1* in HCC cells, *NXT1 *gene expression was silenced through siRNA transfection. As demonstrated in Fig. 8A-8C, *NXT1* siRNA1 notably reduced NXT1 mRNA and protein levels in HCCLM3 cells compared to the siRNA NC group. Meanwhile, *NXT1* siRNA1 remarkably suppressed HCCLM3 cell viability, migration, and invasion compared to the siRNA NC group (Fig. 8D-8H). These results suggested that *NXT1* may function as an oncogene in HCC.

## Discussion

*NXT1* (nuclear transport factor 2-like export factor 1, also known as p15) encodes a protein located in the nuclear envelope, which plays a crucial role in the nuclear export of RNA molecules,^18^ such as mRNAs, transfer RNAs (tRNAs), and small nuclear RNAs (snRNAs), from the nucleus to the cytoplasm. *NXT1* has been identified as a rapidly lethal factor in other diseases, such as MYCN-amplified neuroblastoma.^19^ Additionally, *NXT1* is essential for the nuclear export mediated by CRM1.^20^ CRM1 has been shown to contribute to oncogenesis in various cancers.^21^ Inhibition of CRM1-mediated nuclear export has been demonstrated to enhance the accumulation of mTOR in the nucleus, resulting in decreased protein levels of mTOR and its downstream targets, STAT3 and MMP9, in ovarian cancer cells.^22^ Similarly, the results indicated that downregulation of *NXT1* notably reduces HCC cell proliferation, migration, and invasion, suggesting that *NXT1* may function as an oncogene in HCC. However, the mechanisms by which *NXT1 *regulates HCC progression remain poorly understood. Future studies are necessary to explore the molecular mechanisms underlying its function and to investigate whether *NXT1* exerts its oncogenic effects in HCC cells via a CRM1-dependent pathway.

Furthermore, the results demonstrated that* NXT1 *may potentially act as a potential marker for predicting HCC prognosis. High levels of *NXT1* were associated with poor prognosis of HCC patients. Research has highlighted the crucial role of immune cell infiltration within the TME, demonstrating its close relationship with patient survival and prognosis.^23^ In particular, the infiltration of M1 macrophages within tumor tissue correlates with a more favorable prognosis, whereas a high infiltration of M2 macrophages and Tregs within tumor tissue tends to correlate with worse prognosis of cancer patients.^23^^,^^24^ The results align with these observations, revealing that, compared to the *NXT1*^low^ group, patients in the *NXT1*^high^ group exhibited increased infiltration of M0 macrophages and Tregs, along with a notable decrease in M1 macrophage infiltration. These findings showed that elevated levels of *NXT1* contribute to poor prognostic outcomes in HCC patients, likely due in part to an imbalance of immune cell populations.

Treg cells represent a subset of immunosuppressive cells that inhibit the immune system’s response to tumors and promote IE. Research has indicated that Tregs can be chemoattracted to the TME by specific chemokines, including CCL20 and CXCL5.^25^^,^^26^ In a study conducted by Li et al,^25^ it was demonstrated that DDR1/CXCL5 facilitates the creation of neutrophil extracellular traps, thereby enhancing the immune infiltration of Tregs, which promotes tumor growth and metastasis. Chen et al^26^ discovered that the CCL20-CCR6 axis is responsible for the movement of circulating Tregs into the TME, consequently contributing to disease progression and unfavorable outcomes in HCC patients. The findings indicate a positive correlation between *NXT1* expression and the levels of *CXCL5* and *CCL20 *in HCC. Given this information, it was proposed that *NXT1* may enhance the infiltration of Tregs into the TME by influencing chemokines and their receptors. This mechanism may promote IE, ultimately leading to a worse prognosis in HCC. However, this hypothesis requires further validation in future studies.

Genetic mutations are closely linked to the progression of cancer.^27^ For example, *TP53*, a key tumor suppressor, is frequently mutated in human cancers, rendering its alteration a carcinogenic factor.^27^ To gain deeper insights into the influence of *NXT1* on the progression and prognosis of HCC, the mutation status of genes in the *NXT1*^high^ and *NXT1*^low^ groups was analyzed. The results showed that mutations in the *TP53* gene are the most common in both groups, with a higher mutation rate observed in the *NXT1*^high^ group compared to the *NXT1*^low^ group. These findings showed that elevated expression of *NXT1* correlates with more frequent *TP53* mutations in HCC, implying a potential link between *TP53* mutation and *NXT1* levels in HCC progression, which warrants further investigation in future research.

To summarize, this research for the initial time demonstrated that elevated *NXT1* expression is linked to an unfavorable prognosis and correlates with the presence of various immune cell types and levels of immune checkpoints. Additionally, the results also found that downregulation of *NXT1* notably suppresses HCC cell viability, migration, and invasion, suggesting the oncogenic roles of *NXT1* in HCC. These results imply that *NXT1* may serve as a prognostic marker and a potential therapeutic target for HCC.

Furthermore, there are some limitations in this article. The rates of incidence and mortality for HCC differ significantly among various geographic and ethnic groups. Further research is required to determine whether geographical and ethnic differences can influence the ability of *NXT1 *to predict the prognosis of HCC patients, as well as investigate whether *NXT1* can serve as an independent risk factor for HCC patients. Moreover, the molecular mechanism by which *NXT1* exerts its oncogenic effects in HCC warrants comprehensive investigation in future studies. Meanwhile, the anti-tumor effect of *NXT1* deficiency in HCCwas exploredin vitro; however, the function of *NXT1* in HCCin vivo remains unexamined and requires further investigation. Furthermore, the results showed that HCC patients in the *NXT1*^high^ group exhibited higher levels of 8 immune checkpoints than that in the *NXT1*^low^ group, indicating that they might be more likely to benefit from immunotherapy. It has been demonstrated that hepatitis B virus (HBV), hepatitis C virus (HCV), and nonalcoholic fatty liver disease (NAFLD) are well-established as the predominant etiological drivers of HCC.^28^^,29^ Clinical evidence indicates that HCC patients with different underlying etiologies exhibit distinct responses to ICI therapy.^30^ Patients with HBV-related HCC may benefit more from ICI treatment compared to those with HCV-related HCC.^30^ While these observations suggest a critical role for disease etiology in influencing treatment outcomes, the potential involvement of *NXT1* in this context remains unexplored. Unfortunately, the public databases utilized in this research did not provide clinical annotations of HCC etiologies such as HBV/HCV infection or NAFLD. Consequently, stratifying patients based on their underlying causative factors was not possible, which hindered the evaluation of whether *NXT1* expression differentially influences prognosis or ICI treatment responses across these etiological subgroups. Future studies incorporating well-annotated, etiology-stratified cohorts will be essential to address this question.

## Supplementary Materials

Supplementary Material

## Figures and Tables

**Table 1.** Clinicopathological Characteristics of Liver Hepatocellular Carcinoma Patients from The Cancer Genome Atlas Database

Characteristics		Patients (n = 368)	
		n	%
Gender	Female	119	32.3370
	Male	249	67.6630
Age	<61 years (median)	173	47.0109
	≥61 years (median)	195	52.9891
Race	White	183	49.7283
	Black or African American	17	4.6196
	Asian	157	42.6630
	Unknown	10	2.7174
	American	1	0.2717
Survival time	Long (>5 years)	43	11.6848
	Short (<5 years)	325	88.3152
OS status	Dead	131	35.5978
	Alive	237	64.4022
Stage	I	172	46.7391
	II	85	23.0978
	III	83	22.5543
	IV	4	1.0870
	Unknown	24	6.5217
Grade	G1	55	14.9457
	G2	176	47.8261
	G3	120	32.6087
	G4	12	3.2609
	Unknown	5	1.3587
Pathologic T	T1	182	49.4565
	T2	92	25.0000
	T3	78	21.1957
	T4	13	3.5326
	Unknown	3	0.8152

OS, overall survival.

**Table 2.** Clinicopathological Characteristics of Hepatocellular Carcinoma Patients from International Cancer Genome Consortium-Liver Cancer-RIKEN-Japan Database

Characteristics		Patients (n = 231)	
		n	%
Gender	Female	61	26.41
	Male	170	73.59
Age	<69 years (median)	115	49.78
	≥69 years (median)	116	50.22
Survival time	Long (>5 years)	2	0.87
	Short (<5 years)	229	99.13

## Data Availability

The data that support the findings of this study are available in The Cancer Genome Atlas (TCGA, https://tcga-data.nci.nih.gov/tcga/) database, the International Cancer Genome Consortium (ICGC, https://dcc.icgc.org/) and the Gene Expression Omnibus (GEO, https://www.ncbi.nlm.nih.gov/geo/) database.
