# Supplementary material for: Immune Escape-Related Gene NXT1 as a Potential Prognostic and Therapeutic Target in Hepatocellular Carcinoma
Source: Turk J Gastroenterol. 2025 Aug 25;37(1):98–112. doi: 10.5152/tjg.2025.24193 (PMC12824892; doi:10.5152/tjg.2025.24193)
Supplement: Supplementary Material [file supplementary_material.pdf]

Supplementary Table 1. Immune escape related genes used in this study.

Gene Symbol	Pigs	Det1	Pced1b
B2m	Pigu	Dicer1	Pdcd6ip
Ifngr1	Ppp2r2a	Dnajc13	Pdss2
Jak2	Psmb8	Dot1l	Pi4kb
Socs1	Rb1cc1	Dph5	Pigk
Tap1	Rbm15	Eif3h	Pigt
Tap2	Ric8	Emc2	Pkn2
Tapbp	Rnf31	Emc3	Ppp1ca
Adar	Scaf4	Emc4	Ppp1r8
Atg10	Smg7	Emc6	Ppp2r3c
Atg101	Tab1	F8a	Prkcsh
Cflar	Tab2	Fam58b	Psmb9
Fitm2	Tmem127	Fas	Psmg1
Gpaa1	Tnfrsf1b	Fntb	Ptar1
Gpi1	Ube2g2	Gale	Rce1
Ifngr2	Ube2n	Gls	Rgp1
Ikbkg	Vdac2	H2-K1	Ric1
Irf1	Vps29	Hcfc2	S100pbp
Jak1	Vps35	Hdgfrp2	Sarnp
Otud5	Vps4b	Hexim1	Setd1a
Ptpn2	Wipi2	Hira	Setdb1
Rbck1	Zcchc14	Hspa13	Slc25a32
Stat1	Acad9	Ifnar1	Spcs1
Stat2	Actb	Ifnar2	Srrt
Tnfaip3	Ago2	Ikbkb	Srsf7
Tnfrsf1a	Ahsa1	Ino80	Stoml2
Traf2	Anapc15	Ippk	Susd6
Atg12	Arf6	Irf9	Tbk1
Atg3	Atg14	Jagn1	Tfrc
Atg5	Atg7	Jmjd6	Tgfbr2
Becn1	Atg9a	Kat6a	Tmem208
Chic2	Atp13a1	Klf16	Tradd
Dnttip1	Atxn7l3	Kmt2a	Trpm7
Emc8	BC003331	Lipt2	Ubr5
Erap1	Bcl2l1	Mapk1	Ufc1
Erp44	Bola3	Med16	Ufl1
Fadd	Brat1	Med23	Usp7
Hdac1	Brpf1	Med24	Uxs1
Ist1	Cad	Mgat1	Vps13a
Map3k7	Calr	Mta2	Vps16
Men1	Cep55	N6amt1	Wdr7
Mogs	Chmp5	Ncbp1	Wdr83
Ndufa13	Cox6c	Ndufaf6	Wwp2
Nxt1	Crebbp	Nploc4	Yap1
Otulin	Cul3	Nup188	Zc3h3
Pdia3	Dcp1a	Pcbp2	

Supplementary Table 2. The clinical information of HCC patients.

No.	Age	Gender	Tumor Size (cm×cm)	T	N	M	Clinical stage	Etiology (Alcohol, HBV, HCV, others)
1	55	Male	5.3×4.7	2	0	0	II	HBV
2	61	Male	15×11.5	3	0	0	IIIA	HBV
3	73	Male	2.5×2.0	2	0	0	II	HCV
4	70	Male	1.5×1.5	2	0	0	II	HBV
5	78	Male	9.7×9.0	4	0	0	IIIB	Alcohol
6	67	Female	5.0×4.5	2	0	0	II	No
7	59	Male	3.5×2.2	2	0	0	II	Alcohol
8	55	Male	3.5×3.0	1b	0	0	I	HBV
9	67	Female	7.0×6.5	2	0	0	II	HBV
10	66	Female	2.6×2.9	2	0	0	II	HBV

HBV, hepatitis B virus; HCV, hepatitis C virus.

Supplementary Table 3. Primer sequences for qPCR.

Genes	Forward Primer (5′-3′)	Reverse Primer (5′-3′)
Nuclear Transport Factor 2 Like Export Factor 1 (NXT1)	CTTCCAGCGAGTTCCAAATCA	CAGATGACAACAAGGACCGTG
Glyceraldehyde-3-Phosphate Dehydrogenase (GAPDH)	GAAGGTGAAGGTCGGAGTC	GAAGATGGTGATGGGATTTC

Supplementary Table 4. siRNA sequences.

Genes		Sequences (5′-3′)
Nuclear Transport Factor 2 Like Export Factor 1 (NXT1) siRNA1	sense	GAUCGCAAGUGACUGCUUC
	antisense	GAAGCAGUCACUUGCGAUC
NXT1 siRNA2	sense	CCUCGACUCUCAAGGAUGU
	antisense	ACAUCCUUGAGAGUCGAGG
NXT1 siRNA3	sense	GCGAGUUCCAAAUCAGCGU
	antisense	ACGCUGAUUUGGAACUCGC
Negative control	sense	UUCUCCGAACGUGUCACGU
	antisense	ACGUGACACGUUCGGAGAA

Supplementary Table 5. Gene Ontology (GO) enrichment analysis.

https://docs.google.com/spreadsheets/d/1asVV7t8WCwZ_ZeJxLgwaPZtxjzis_KlK4Cr9-lKdlUU/edit?usp=sharing

Supplementary Table 6. Kyoto Encyclopedia of Genes and Genomes (KEGG) enrichment analysis.

ID	Description	BgRatio	pvalue	p.adjust	qvalue	geneID	Count
hsa05169	Epstein-Barr virus infection	202/8292	0.0002699	0.0040826	0.0029952	TAP2/TRADD/TAPBP	3
hsa05170	Human immunodeficiency virus 1 infection	212/8292	0.0003113	0.0040826	0.0029952	TAP2/TRADD/TAPBP	3
hsa05163	Human cytomegalovirus infection	225/8292	0.0003711	0.0040826	0.0029952	TAP2/TRADD/TAPBP	3
hsa04612	Antigen processing and presentation	78/8292	0.0012787	0.0105493	0.0077395	TAP2/TAPBP	2
hsa05164	Influenza A	171/8292	0.0060056	0.0396371	0.0290798	NXT1/TRADD	2
hsa00563	Glycosylphosphatidylinositol (GPI)-anchor biosynthesis	26/8292	0.018672	0.1026962	0.0753433	PIGT	1
hsa05340	Primary immunodeficiency	38/8292	0.0271914	0.120401	0.0883325	TAP2	1
hsa02010	ABC transporters	45/8292	0.0321325	0.120401	0.0883325	TAP2	1
hsa03050	Proteasome	46/8292	0.0328366	0.120401	0.0883325	PSMB9	1
hsa04920	Adipocytokine signaling pathway	69/8292	0.0489149	0.1509072	0.1107134	TRADD	1
